# Work‐Related Quality of Life and Well‐Being of Social Care Workers in the Disability Sector in Ireland

**DOI:** 10.1111/jar.70087

**Published:** 2025-06-19

**Authors:** Victoria Hogan, Martin Power, Michael Hogan, Margaret Hodgins

**Affiliations:** ^1^ Discipline of Health Promotion, School of Health Sciences University of Galway Galway Ireland; ^2^ School of Psychology University of Galway Galway Ireland

**Keywords:** disability sector, organisational constraints, quality of working life, social care workers, turnover intention, well‐being, workload

## Abstract

**Background:**

Scant attention has been paid to the quality of working life of social care workers in Ireland. This study sought to characterise both the quality of working life and the well‐being of social care workers in the disability sector.

**Method:**

A cross‐sectional survey of social care workers (*n* = 307) was conducted. Measures of work‐related quality of life, well‐being, organisational constraints, workload and turnover intention were included in the survey.

**Results:**

Experiencing higher organisational constraints, longer work hours and higher workload were related to lower quality of working life, *F*(9,242) = 24.953, *p* < 0.001. Higher organisational constraints and higher workload also negatively influenced well‐being, *F*(9,240) = 11.494, *p* < 0.001. Linear regression indicated that higher turnover intention was influenced by lower quality of working life, *F*(1, 279) = 306.319, *p* < 0.001.

**Conclusions:**

The results indicate the importance of the influence of work‐related factors on both quality of working life and well‐being in social care workers in disability services in Ireland.

**Summary**

- In this study, social care workers in the disability sector in Ireland reported lower levels of well‐being and work‐related quality of life in comparison with the levels of well‐being generally experienced by health care workers.
- Experiencing difficulties in daily work due to organisational constraints, a heavy workload and long work hours negatively influenced work‐related quality of life.
- Experiencing difficulties in daily work due to organisational constraints and heavy workload negatively influenced well‐being.
- Having a lower quality of work life meant that social care workers were more likely to want to leave their job.

## 1 Introduction

According to Silarova et al. (2022) it is vital that we understand how to make social care work attractive as a profession in order to ensure its sustainability into the future. The quality of work life construct can be useful in this context as it provides a set of measures to help diagnose problematic workplace issues and identify strategies to improve working life for both the benefit of staff and service users (Martel and Dupuis 2006). Importantly, quality of work life can be measured as a multi‐dimensional construct and thus may provide more information to employers than uni‐dimensional analyses of related constructs such as job satisfaction, job involvement, work‐related stress and so forth. While a number of studies have examined the quality of work life of social care workers across European countries and the UK (see Silarova et al. 2022 for a review), no study to date has examined the work‐related quality of life (WrQoL) of Irish social care workers. Given differences in healthcare systems, workforce composition, regulation and accreditation across countries, there is a need to investigate WrQoL specifically within the Irish social care system and national context.

### 1.1 WrQoL and Well‐Being

There is no consensus on how to define WrQoL (Sirgy et al. 2001) and numerous definitions of WrQoL have been employed in adult social care research to date (Silarova et al. 2022). Reviews of the construct have identified 33 possible components (e.g., Easton and Van Laar 2018; Wallace et al. 2007); however, Silarova et al. (2022) in their review of the adult social care literature propose six primary components to WrQoL: organisational characteristics, job characteristics, mental well‐being/health, physical well‐being/health, spill‐over from work to home and professional identity. At the same time, Silarova et al. (2022) noted that no study to date has examined all of these components. Wang et al. (2023) in their review of instruments designed to measure WrQoL in healthcare and social service staff reported that most instruments examine work conditions, job satisfaction, work‐related stress, relationship/balance issues and career progression. To date, questionnaire instruments generally include measures of work conditions, job satisfaction and Stress at Work (SAW), and most include other unique measures related to quality of work life that vary from one instrument to the next (Wang et al. 2023). In this study, we adopt the Van Laar et al. (2007) model of WrQoL, which proposes a six‐factor structure, composed of (1) General Well‐Being (GWB), (2) Home–Work Interface (HWI), (3) Job and Career Satisfaction (JCS), (4) Control at Work (CAW), (5) Working Conditions (WCS) and (6) SAW. The Van Laar et al. (2007) model and measurement instrument of WrQoL was chosen for this study as it was specifically designed for use with healthcare workers (HCW) and has previously been employed in studies examining WrQoL in social care workers for example, McFadden et al. (2020); Engström et al. (2023) and other allied healthcare professionals, including occupational therapists (Hogan et al. 2023) and nursing home staff (Rai 2013).

The shortage of health and social care workers is a recognised problem both internationally (McGill Nursing Collaborative 2019) and within Ireland (Department of Children, Equality, Disability, Integration and Youth 2023; Keegan et al. 2022). The landscape for HCWs, including social care workers both pre and post‐Covid is challenging (McFadden et al. 2021), with high stress levels reported and lower levels of well‐being (Kinman 2021). In a national study by McFadden et al. (2021) examining WrQoL in allied HCWs in the UK during the Covid pandemic, approximately one third of social care workers reported low WrQoL. WrQoL has been linked to higher turnover intention across several healthcare groups, including nurses (Kaddourah et al. 2018; Poku et al. 2022), occupational therapists (Hogan et al. 2023), hospital employees (Shukla et al. 2017) and social care workers (Engström et al. 2023). In a study which employed the WrQoL questionnaire instrument, Engström et al. (2023) examined turnover intentions and the influence of WrQoL on nurses and social care workers. Within the study, 22% of social care workers indicated that they intended to leave both their profession and their workplace. WrQoL factors significantly related to turnover intention included higher levels of work‐related stress, poorer work‐life integration and lower levels of job/career satisfaction. McFadden et al. (2021) have reported that personal factors (i.e., ethnicity and relationship status) were related to WrQoL in allied HCWs, while Hogan et al. (2023) reported that work‐related characteristics (i.e., workload and organisational constraints) were related to WrQoL in Irish occupational therapists. Social care workers are regarded as being particularly vulnerable to experiencing low WrQoL due to a combination of work and situational factors, including unfavourable WCS, low pay, and the emotional toll associated with the work, leading to both physical and mental stress and burnout (Hussein et al. 2022; Towers et al. 2022).

### 1.2 Social Care in Ireland

Social care workers in Ireland operate in a mixed economy of care that includes public, private and voluntary/charitable providers, with the latter often partially or fully funded from the public purse by the Health Service Executive (Byrne 2016). The mixed economy approach varies significantly by sector, with private providers dominating the provision of children's residential services and care of older people, while care for individuals with disabilities is largely supplied by voluntary/charitable organisations (Lyons 2017; Mulkeen 2016). This approach to social care in Ireland has been shaped by the historical influence of the Catholic Church in health and education, a consequently residual attitude to state welfare and an arms‐length approach by the state to service provision (Harvey 2007; Moran 2013).

These historical and mixed economy influences have been influential in shaping social care work in Ireland, with recognition of social care work as a distinct profession only gaining traction with the inclusion of social care worker as a professional title in the 2005 Health and Social Care Professionals Act. However, a state register for social care workers under the remit of CORU, Ireland's multi‐professional health and social care regulator, did not open until November 2023 and there is a 2 year‐grandparenting period (CORU 2023). As such, it will be the end of 2025 before a definitive figure can be put on the number of social care workers in Ireland (Hanrahan 2023). The near 20 year wait between the Act and a register opening can be attributed to the complexities of regulating a previously unregulated and diverse grouping that operates across many sectors, with multiple entry routes into social care work (CORU 2019). Indeed, the term social care worker was created in the mid‐1990s in an effort to encompass the diversity of worker roles under a common term, but it has not been widely endorsed (Lyons 2017; Power and D'Arcy 2017). Moreover, social care workers feel that social care work is poorly understood, remains the poor relation among other professions, and is almost unknown among the general public (Power and Dashdondog 2023).

The absence of a commonly accepted title and the legacy of an organisational rather than professional led evolution of social care work continues to exert a substantial influence. While the workforce is predominately female, job titles and roles and responsibilities vary significantly within and between sectors and organisations (Power and D'Arcy 2017). Moreover, pay and WCS are felt to be inadequate, especially in for‐profit providers, while long unsocial hours and a general lack of respect have been identified as other key challenges for recruitment and retention (Power and Burke 2021). This is often compounded by concerns around the extent of risks to be managed in day‐to‐day practice, inadequate staffing and very limited progression and career pathways, with an all too common glass ceiling of social care leader (Power and Dashdondog 2023). Thus, it is unsurprising that many view social care work as merely a means to another career or profession rather than an end in itself (Power and Dashdondog 2023).

### 1.3 Aims

Silarova et al. (2022) have identified 68 studies to date that have examined WrQoL among workers in adult social care, noting a lack of agreement on how to define WrQoL and its measurement. The results from the systematic review by Wang et al. (2023) examining instruments employed to measure WrQoL among social services workers support the lack of consistency in approaches to measurement, identifying 13 different instruments that have been employed to date. Considering that over 30 WrQoL studies have been conducted across the EU and eight in the UK/England (Silarova et al. 2022), the lack of attention to the issue of WrQoL in the Irish setting is of concern. Therefore, this study aims to characterise the well‐being and WrQoL of social care workers in the disability sector in Ireland using a questionnaire measure of WrQoL designed for use with HCWs and previously employed in examinations of this issue with social care workers in other European countries (Engström et al. 2023; McFadden et al. 2021). In particular, the influence of personal and workplace characteristics on WrQoL will be investigated in conjunction with an examination of the relationship between WrQoL and turnover intentions.

## 2 Methods

### 2.1 Participants

National figures for the number of social care workers in Ireland are not available and there is no national association of social care workers through which the questionnaire could be distributed. In the current study, social care workers, who were working in the Disability Services Sector were designated as the target population and were approached through a network of national contacts available to the research team. A number of gatekeepers agreed to distribute the questionnaire to workers in the Disability Services Sector. In addition, the study was advertised through social media. Although formal population figures for social care workers in the disability sector are not available, an approximate figure of 4000 in the sector is likely (Health Service Executive 2024). In total 308 questionnaires were completed, of which 307 were usable, representing 8% of the estimated population in the disability sector.

### 2.2 Procedure

This study employed a cross‐sectional, multi‐variate questionnaire survey design. A link to an electronic survey questionnaire in Microsoft Forms was sent to the gatekeepers for distribution to participants. The email invitation to participate in the study included a participant information leaflet which provided a study overview. Data was collected in June–July 2022.

### 2.3 Measures

A questionnaire was designed for the study with reference to the extant literature. The questionnaire included three sections, which examined (1) demographics of the sample, (2) work characteristics and (3) validated measures of WrQoL, turnover intentions, well‐being, quantitative workload and organisational constraints. One open‐ended question was included to allow participants to comment on their quality of working life. Additional details on the survey measures employed are provided below.

- Demographic questions: Age, gender, ethnicity, marital status and caring responsibilities were included.
- Work characteristics: 15 questions were employed to examine features of work, which included professional area of work, location in country, sector, job tenure and role, working hours/overtime, sick leave, redeployment.
- WrQoL: Measured using the Quality of Working Life Scale (Van Laar et al. 2007). This scale includes six factors: (1) GWB (six items), (2) HWI (three items), (3) JCS (six items), (4) CAW (three items), (5) WCS (three items) and (6) SAW (two items). Responses for all items employ a five‐point Likert scale ranging from ‘Strongly Disagree’ to ‘Strongly Agree’. Scoring instructions by Easton and Van Laar (2018) were followed to compute factor scores and an overall score for the measure. Higher scores reflect better quality of work life.
- Turnover intentions: The Turnover Intention Scale (Michaels and Spector 1982) was employed. This measure includes three items (e.g., ‘I often seriously consider leaving my current job’). Responses are scored on a six‐point Likert scale ranging from ‘strongly disagree’ to ‘strongly agree’. Higher scores reflect greater turnover intentions.
- Well‐being: GWB was measured using the WHO‐5 Well‐being Index (WHO‐5; World Health Organisation 1998). This measure employs five items (e.g., ‘I have felt cheerful and in good spirits’). Responses are provided using a five‐point Likert scale ranging from ‘at no time’ to ‘all of the time’. Higher scores indicate better well‐being.
- Workload: The Quantitative Workload Inventory (Spector and Jex 1998) was employed, which consists of five questions. Respondents are asked to indicate how often different situations occur (e.g., how often do you have to do more work than you can do well?). Responses are provided on a five‐point Likert scale ranging from ‘less than once per month’ to ‘several times per day’. Higher scores reflect higher levels of workload.
- Organisational Constraints: The Organisational Constraints Scale (Spector and Jex 1998) was employed, which is composed of 11 items. The items measure how difficult work is to do because of constraints (e.g., ‘Interruptions by other people’). Responses are provided on a five‐point Likert scale ranging from ‘less than once per month’ to ‘several times per day’. Higher scores reflect a higher level of constraints.

### 2.4 Statistical Analyses

Descriptive and inferential statistics were conducted on the survey data, including the calculation of means and standard deviations for continuous variables, and frequencies and percentages for categorical level data. Regression analyses were employed for the purpose of addressing the research objectives, which were to determine the relationship between personal and work‐related variables on WrQoL and well‐being and to determine the relationship between WrQoL and turnover intentions. Cronbach alpha was calculated to assess scale reliability of the measures employed in the survey questionnaire. Table 2 presents the Cronbach alpha values for the WrQoL sub‐scales and the WHO‐5, QWI and OC measures. All measures employed demonstrated good to very good Cronbach alpha reliability with all scales exceeding 0.7. Quantitative data analysis was conducted using SPSS, Version 29.

### 2.5 Content Analysis

Inductive category coding (Mayring 2000) was employed to analyse the responses to the open‐ended question. This form of conventional content analysis is considered appropriate when the study aim is to describe a phenomenon (Hsieh and Shannon 2005), which in the case of this study is WrQoL.

### 2.6 Ethical Approval

Ethical approval for the study was provided in March 2022 by the University of Galway Research Ethics Committee. Informed consent was sought from all study participants through a mandatory informed consent question at the start of the questionnaire survey.

## 3 Results

### 3.1 Quantitative Results

Table 1 presents the demographic and work characteristics of the sample. The majority of the sample was female (*n* = 278, 91%), aged less than 40 years (61%) and almost half (49%) had children. Over half (54%) had completed undergraduate studies, and the majority were in full‐time employment (85%) and had permanent positions (95%).

**TABLE 1:** Demographic and work characteristics of participants.

Variable	*N* = 307	Percentage
Age		
18–29	74	24
30–39	114	37
40–49	83	27
50–59	29	10
60–65	6	2
Marital status		
Single	90	30
Married	132	44
Co‐habiting	68	22
Divorced/separated	14	4
Children		
Yes	149	49
No	158	51
Qualification		
Secondary school	1	0.5
Undergraduate	164	54
Postgraduate	137	45
PhD	1	0.5
Employment status		
Full time	261	85
Part time	46	15
Job permanence		
Permanent	289	95
Temporary	10	3
Agency	5	1.5
Independent (self‐employed)	1	0.5
Sector		
Public	142	48
Private	76	25
Voluntary/community/other	79	27
Years of work experience		
< 5	91	29
6–10	85	28
11–20	85	28
21–30	38	12
> 30	8	3

Hours worked	Mean	(SD)
All workers	38.38	8.9
Full time workers	40.38	7.78
Part time workers	27.06	5.96

**FIGURE 1:**
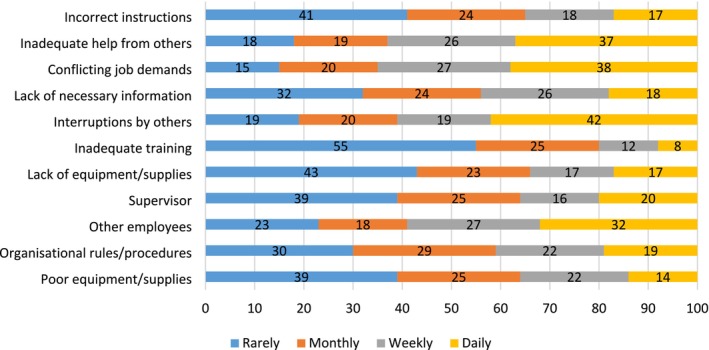
The distribution of responses to the 11 questions of the Organisational Constraints Scale by participants. Numbers within the figure indicate the percentage of the sample who reported the constraint. Colour coding of the bars indicates the frequency at which each organisational constraint was experienced. Blue indicates that a constraint was experienced rarely, orange indicates that a constraint was experienced on a monthly basis, grey indicates that a constraint was experienced weekly and yellow indicates that a constraint was experienced daily. For example, 42% of participants reported being interrupted by others on a daily basis whereas only 8% of participants reported inadequate training being a daily constraint.

The average scores and standard deviations for the WrQoL sub‐scales and overall measure are presented in Table 2. These scores were compared to published normative data for the WrQoL measure (Easton and Van Laar 2018), which were generated for HCWs in the UK. The overall score and sub‐scale scores are all towards the lower end of the distribution. For example, the average score of 2.85 on the CAW sub‐scale indicates that the score is within the 20th percentile, which means that the average CAW score in this study is equal to or higher than 20% of HCWs in the UK. The overall score on the WrQoL measure is within the lowest percentile that is, between the 10th–20th percentile. Scores for the QWI and OCS were also compared to normative data and in both cases the average scores of 18.71 and 27.61 were higher than the norms, indicating a higher level of reported quantitative work intensity and organisational constraints. Figure 1 presents the distribution of the responses to the Organisational Constraints questionnaire items, presented as percentages. Almost one third (32%) of participants indicated that other workers constrained their work activity on a daily basis, while over one third indicated that inadequate help (37%), conflicting job demands (38%) and interruptions by others (42%) constrained their work daily. The WHO‐5 score (43.44) was compared to the cut‐off threshold of 50, which is the level under which scores indicate lower well‐being (Topp et al. 2015). While there was no comparative normative data for the Turnover Intentions measure, descriptive statistics demonstrate that 71% agreed that they were considering resigning from their jobs, 61% were looking for other jobs, and just over half (51%) intended to quit.

**TABLE 2:** Means, standard deviations and Cronbach alphas on questionnaire measures.

	Mean	SD	Normative data comparison	Cronbach's alpha
Work‐related quality of life	2.85	0.68	10th to 20th percentile	
General Well‐being Factor (GWB)	3.09	0.79	20th to 25th percentile	0.875
2 Home–Work Interface (HWI)	2.92	1.01	20th to 25th percentile	0.801
3 Job Career Satisfaction (JCS)	3.14	0.78	25th percentile	0.797
4 Control at Work (CAW)	2.85	0.89	20th percentile	0.729
5 Stress at Work (SAW)^a^	2.05	0.87	20th–30th percentile	
6 Working Conditions (WCS)	3.02	0.91	25th–30th percentile	0.754
Turnover intention	12.35	5.21		0.913
WHO‐5 well‐being	43.44	21.15		0.885
Organisational Constraints^b^	27.61	9.92	Higher than norm	
Quantitative Workload Inventory	18.71	5.63	Higher than norm	0.915

^a^ Cronbach alpha not calculated as only two items in scale.

^b^ Calculation of Cronbach alpha is not advised (Spector and Jex 1998).

#### 3.1.1 Regression Analysis

For the first regression model examining the relationship between personal and work‐related variables on WrQoL, in the first instance Pearson's correlation analysis was conducted on the predictor variables included in the model. This indicated that a number of the variables were correlated, however, these ranged from weak to moderate level correlations. Among the predictor variables for example, QWI was positively correlated with OCS, *r* = 0.642, *p* < 0.001. In addition, variance inflation factors were checked for all predictor variables and ranged from 1.050 to 1.784, demonstrating that multi‐collinearity was not a significant issue. The variables were then entered in three blocks, with the personal variables entered as the first block (age, marital status, children) and the work‐related variables entered in the second block (full‐time/part‐time status, years' work experience, sector, hours per week) and the QWI and OCS variables were entered in the third block. A significant model was produced, *F*(9,242) = 24.953, *p* < 0.001, that explained 46% of the variance (Adjusted *R* ^2^ = 0.462). The standardised regression coefficients and significance levels for the variables included in the final model are presented in Table 3. A second multiple regression analysis was conducted to determine the influence of personal and work‐related variables on well‐being. The variables were entered as per the approach for WrQoL. A significant model was produced, *F*(9,240) = 11.494, *p* < 0.001, that explained 28% of the variance (Adjusted *R* ^2^ = 0.275). Table 3 presents the standardised regression coefficients and significance levels for the predictor variables included in the final model. Linear regression was conducted to examine the influence of WrQoL on turnover intention. The model was significant, *F*(1, 279) = 306.319, *p* < 0.001, and explained 52% of the variance (Adjusted *R* ^2^ = 0.522), *B* = −5.448, *B* SE = 0.311, *β* = −0.723.

**TABLE 3:** Multiple linear regression analyses for the outcome variables WrQoL and well‐being.

Variable	WrQoL		Well‐being	
	*β* (standardised beta value)	*p*	*β* (standardised beta value)	*p*
Age	−0.011	0.855	0.128	0.067
Marital status	−0.039	0.410	0.011	0.840
Children	−0.020	0.679	0.039	0.496
Full time/part time	−0.071	0.174	0.057	0.348
Work experience	−0.035	0.561	−0.052	0.456
Sector	0.035	0.467	0.032	0.558
Work hours	−0.141	0.007*	−0.051	0.470
Quantitative Workload	−0.171	0.006*	−0.208	0.004*
Organisational Constraints	−0.549	< 0.001*	−0.339	< 0.001*

* Denotes statistical significance.

### 3.2 Open Question

Of the 307 respondents, 88 provided additional comments, illustrating how aspects of the work environment impacted quality of working life. Notably, only one participant (86) described their work positively, stating ‘I love my job. Most of the time’. Stress‐inducing work conditions was a prominent theme, with multiple stressors such as workload increases, staff shortages, high responsibility and low salary referred to. For example, ‘staffing crisis, under qualified and uninterested staff, Covid issues and working in an extremely difficult and dangerous work environment have made me change from a social care worker who loved my job to a defeated and injured and permanently stressed social care leader’ (82). Participants variously described negative outcomes as a result of these work conditions, including experiencing fatigue, stress, burnout, being overwhelmed and a lack of rest and recovery time. For example, one participant noted that they were ‘Feeling extremely overwhelmed more and more, only for my colleagues in my centre and our team work I feel I might not be able to do this role long term with things as they are currently’ (71). Social care work was described as intense, physically and mentally draining, even dangerous at times and while this was accepted as the nature of the job, the lack of support and not being listened to by management was how quality of working life was eroded. As noted by one respondent, ‘*Regular staff are going beyond their job title everyday with little or no support from management with the attitude of “*just get on with it”’ (32).

Work‐life balance issues were also another prominent theme that respondents identified as causing stress, exhaustion and potentially leading to isolation and loneliness. Work‐life balance issues were seen to be a function of poor management practices in relation to shift work. Respondents identified long shifts, inconsistent rostering and inadequate rest breaks between shifts as punitive shift patterns. Relatedly, waking nights and sleepovers that ‘don't count as hours worked’ (40) were particularly difficult for staff impacting on the quality of family life and on individual health and well‐being. Management were seen to ‘*not abide by the Working Time Act*’ (59) and as such respondents referred to poor and uncaring management practices. Conversely, some respondents highlighted attention from management to rostering, and/or being able to negotiate part‐time hours, which was seen as positive; ‘I work 40 h a fortnight. This does not include sleepovers. I reduced my hours so I can get the work life balance that is needed. Before I reduced my contracted hours I was extremely unhappy and stressed’. (40).

Workload challenges and poor management intersected with the reported experience of social care work post‐Covid. Respondents mentioned not having breaks, being expected to be responsive at all times to client needs, and being on call or texted out of hours, for example: ‘Expectation of always being available is a bad culture of social care, Covid has created the virtual manager which is very disheartening for front line workers’ (56). So even when not in work, social care working practices create a spillover effect, impacting negatively on quality of life.

## 4 Discussion

Given that the WrQoL of social care workers in Ireland has not been examined to date, this study sought in the first instance to characterise WrQoL and well‐being in social care workers in the disability sector. A second objective was to investigate the relationship between personal and work‐related variables and both WrQoL and well‐being, similar to the research conducted by McFadden et al. (2021) in their national investigation of WrQoL in HCWs in the UK. The relationship between WrQoL and turnover intention was also examined. A number of work‐related factors were highlighted as significant in the regression analyses, that is, work hours, workload and organisational constraints in the case of WrQoL, and workload and organisational constraints in the case of well‐being. In keeping with the extant literature on turnover intentions among HCWs, the results of the regression analysis also revealed a positive relationship between lower WrQoL and higher turnover intention.

The mean WrQoL score (2.85) in this study equates to only the 10th–20th percentile of normative scores on the WrQoL measure (Van Laar et al. 2007), comparing unfavourably with previous research. For example, it is lower than the mean score (3.7) reported by Engström et al. (2023) in their study of social care workers and nurses in Sweden, the mean score (3.4) reported by McFadden et al. (2021) in their study of HCWs, including social care workers in the UK, and also the mean score (3.16) reported by Hogan et al. (2023) in their study of occupational therapists in Ireland. The scores on the six components of the WrQoL measure were also at the lower end of the distribution, with the scores ranging from the 20th to the 30th percentile of the normative scores. The qualitative responses where participants described their WrQoL further reinforce and provide additional context to explain the low scores on the SAW and HWI subscales of the WrQoL measure.

The results from the regression analysis demonstrated that longer working hours alongside higher levels of organisational constraints and higher workload significantly predicted lower WrQoL. Although previous research has noted that personal variables can influence WrQoL in social care workers (McFadden et al. 2021), personal variables were not found to be significant predictors in this study. Reported work hours in this study ranged from 17 to 80 h a week, with an average of 38 h per week. Only a minority of participants (6%) reported working over 50 h per week, and average hours worked also depended on the full‐time versus part‐time employment status of workers, with mean working hours of 40 and 27 h, respectively. While working hours for the majority are within the normal range, the mean score on the Quantitative Workload Inventory (18.71) is higher than the available norm (16.5), which implies a high level of demand upon social care workers during their shifts. Towers et al. (2022) reported that heavy workloads experienced by social care workers impede service delivery, with a lack of time and resources available to support the people that they care for. The mean score (27.61) on the organisational constraints measure is also higher than the norm (21.3) and is higher in comparison with the mean score (25.35) reported by occupational therapists working largely in the public health sector in Ireland (Hogan et al. 2023). The organisational constraint score in this study can be further understood by reference to particular constraints reported by social care workers in their daily work. In particular, the analysis of the individual items on the Organisational Constraint measure indicates that social care workers frequently experienced constraints related to co‐workers, a lack of help and experiencing frequent interruptions, with conflicting work demands being the most frequently identified (42%) daily constraint.

The average score of 43.44 on the WHO‐5 well‐being instrument is considerably lower than the threshold score of 50 which is considered to denote low well‐being. This score also compares unfavourably with the average score (52) reported for the general public in Ireland by Guzman et al. (2020) in their Green‐Covid survey and is lower than the average score (53.5) reported by Hogan et al. (2023) for occupational therapists. This low level of average well‐being among social care workers in the disability sector concurs with that of McFadden et al. (2021) who reported lower well‐being in social workers when compared with the general public in their UK based study of allied care professionals. However, this comparison must be interpreted with caution due to the use of two different instruments to measure well‐being; in this study the WHO‐5 was employed while McFadden et al. (2021) used the Short Warwick Edinburgh Mental Well‐being Scale. Nevertheless, the low level of well‐being among social care workers in the disability sector in Ireland is concerning. The low level of well‐being reported can be further understood by reference to the work context described in the qualitative feedback, indicating stressful WCS, in conjunction with lack of management support, contributing to day‐to‐day difficulties for workers. Notably, in the qualitative responses increased work demands were seen to be caused by staff shortages, which would potentially explain why lack of help from others was identified by over a third of respondents as a daily constraint. Certainly, previous surveys of social care workers in the Irish disability sector (*n* = 195) have found that only slightly over one quarter (25.6%) felt there were ‘generally sufficient numbers of staff for the work involved’ (Power and Dashdondog 2023). With regards to stress, Ryan et al. (2019) in their scoping review of work‐related stress and well‐being among direct care workers in intellectual disability services note that stress and strain is experienced within this sector globally and that occupational factors including excessive work demands, low control and low support are related to higher levels of work‐related stress.

While work hours were not related to well‐being in this study, both organisational constraints and quantitative workload were both significant predictors of well‐being in the regression analysis. This finding is partially consistent with that of Hogan et al. (2023) who reported that higher organisational constraints was predictive of lower well‐being in their study of occupational therapists in Ireland. The finding that high workload as denoted by the QWI score (18.71) negatively influenced well‐being is consistent with the qualitative descriptions provided, which indicate that the work is ‘intense’ and ‘physically and mentally draining’. Notably, organisational constraints was the strongest predictor in both the WrQoL and the well‐being regression models. This findings is consistent with Pindek and Spector (2016), who have found that organisational constraints are linked to both physical and psychological strain and predict both strain and well‐being outcomes, even when other stressors have been controlled for. These findings highlight the need for further investigation of the nature and impact of organisational constraints, with a particular focus on how specific constraints can be reduced in efforts to enhance WrQoL and well‐being.

The level of turnover intentions in this study is much higher than that reported by Engström et al. (2023). In this study, 71% of respondents indicated that they had considered resigning from their position, in comparison with 22% of social care workers and 23% of nurses in the Swedish study. Furthermore, just over half (51%) of social care workers in this study reported that they intended to quit, and 61% indicated that they had started to look for other jobs. These rates of turnover intention compare unfavourably with the rate of turnover intention among occupational therapists in Ireland, where Hogan et al. (2023), employing the same turnover intention measure reported that 46% of occupational therapists had considered resigning, while a third reported that they intended to quit. The comparison between this study and Engström et al. (2023) must be interpreted with caution due to the use of different measures of turnover intention. Indeed, the Engström et al. (2023) study differentiated between intention to quit one's current job and intention to leave the profession, offering a helpful insight into the threat to social care services due to turnover and movement towards other professions, whereas our study examined whether social care workers were considering leaving or if they had taken actions to source alternative employment. However, unlike Engström et al. (2023), it is unclear from our findings whether or not a career change in particular was planned. Nonetheless, the level of turnover intentions among Irish social care workers in the current study is worrying, and potentially presents a significant challenge to the provision of services in the disability sector. Poon et al. (2022) previously noted in their review of turnover intention among HCWs that work and organisational factors have been significant influencing factors both before and after the Covid‐19 pandemic. The regression analysis in this study demonstrated the significant influence of WrQoL on turnover intentions.

## 5 Strengths and Limitations

This is the first examination of WrQoL in the social care sector in Ireland, which is timely as social care work, including job roles and the nature of work differ across countries (Silarova et al. 2022). This study employed a widely used model and measure of WrQoL (Van Laar et al. 2007) that was designed specifically for measuring WrQoL in healthcare populations, allowing direct comparison of the study findings to other allied healthcare groups, and to previous studies where the same measure was employed with social care workers (e.g., Engström et al. 2023; McFadden et al. 2021; Rai 2013). The majority of studies to date investigating WrQoL in social care workers have relied on a variety of constructs related to WrQoL for example, job satisfaction, work‐related stress and so forth, but few to date have measured the construct itself (Silarova et al. 2022). Wang et al. (2023) recommend the WrQoL (Easton and Van Laar 2018) measure for use with social care groups because it has good validity, is theoretically driven and is easy to interpret.

At the same time, limitations in this study include potential bias in the sample surveyed, the lack of availability of population size data, and the reliance on approximate estimates of the number of social care workers within the disability sector in Ireland. The potential bias in the sample must be acknowledged. It contrasts considerably with UK figures, based on the recent survey of 7233 adult social care workers (Blake et al. 2024), who were older, less likely to have children and less likely to have attained graduate status. At the same time, the representativeness of the current study sample in the Irish context is also unknown, given that in the absence of a professional body to help with the distribution of the questionnaire, convenience sampling was employed. Social care work has a narrow definition in Ireland and therefore comparisons with other jurisdictions require caution. Additionally, the high number of graduates in this study (99%) is likely to be influenced by the fact that social care work education programmes have been extremely popular in Ireland, especially against the long‐awaited backdrop of state registration and regulation. At the same time, there is a tension within the social care workforce around qualified/unqualified and thus the education levels reflected in studies may not fully reflect the makeup of the workforce (Power and Dashdondog 2023). Due to the reliance of the research team on a number of gatekeepers to distribute the questionnaire to their colleagues and networks, it was not possible to accurately record the number of distributed questionnaires; therefore, a response rate to this questionnaire survey could not be generated; instead, the study presents the proportion of participants as a percentage of the overall population of social care workers in the disability sector in Ireland. The sample achieved in this study was female‐dominated; therefore, it may not be fully representative of the views of male social care workers. It is also worth noting that COVID‐19 is a likely confounder. Data was collected for this study when restrictions had been lifted, although a spike had occurred 3 months previously. Other WrQoL studies also took place during or after COVID (e.g., McFadden et al. 2020) where health care services were impacted differently. As such, while an acknowledged influential factor, the nature of this influence in the context of different jurisdictions is not known and should be considered in the interpretation of findings. Finally, the responses to the open‐ended question reflect only the views of a sub‐sample of the respondents to the survey overall.

## 6 Implications for Policy, Practice and Research

The low levels of WrQoL and well‐being coupled with the high turnover intention reported by participants in this study are concerning and have implications for the sustainability of the social care work force in the disability sector and service delivery. The results suggest that concrete actions are required to improve both well‐being and WrQoL among social care workers in the disability sector. Ideally, as noted by Towers et al. (2022) more resources, more time and more staff would greatly benefit existing staff and operations, although this may not be achievable; therefore, they recommended practical actions that can be implemented, including but not limited to: the provision of predictable shift patterns, mental health first aiders, better holiday allowances and means of contact for lone staff to combat loneliness for example, Zoom calls. Furthermore, in light of the findings from this study, the adoption of the principles of ‘practice management’ (Mansell et al. 2004) as a means of support for both the workers and the individuals served is recommended, as studies demonstrate positive outcomes for workers including higher job satisfaction (Deveau and McGill 2016), higher engagement (Beadle‐Brown et al. 2015), lower stress and greater trust in management and more positive teamwork experiences (Deveau and McGill 2016). The results from this study suggest that a range of such practical measures are required and that intervention studies are now needed to evaluate the impact of such changes in both the short‐ and long‐term. While this study has provided some initial baseline data that will be of use to Irish policymakers, a single study is not sufficient to elucidate the full complexity of social work experience in Ireland, therefore, additional studies are necessary at the national level in order to fully understand the experience of social care workers. Going forward, we support the recommendation by Silarova et al. (2022) that future studies employ theoretically driven measures that are suitable for use with social care workers, such as the WrQoL measure (Easton and Van Laar 2018), thus improving comparability across studies.

## 7 Conclusions

The results indicate the importance of the influence of work‐related factors on both quality of working life and well‐being in social care workers working in disability services in Ireland. The study findings add to the limited evidence base available on quality of work life among social care workers in Europe and can be used to inform changes to policy and practice in the Irish context.

## Ethics Statement

Ethical approval for this study was granted by the University of Galway, Research Ethics Committee (Reference Number: 2022.03.007, March 2022).

## Consent

The authors have nothing to report.

## Conflicts of Interest

The authors declare no conflicts of interest.

## Data Availability

The data that support the findings of this study are available on request from the corresponding author. The data are not publicly available due to privacy or ethical restrictions.
